# A workflow for the integrative transcriptomic description of molecular pathology and the suggestion of normalizing compounds, exemplified by Parkinson’s disease

**DOI:** 10.1038/s41598-018-25754-5

**Published:** 2018-05-21

**Authors:** Mohamed Hamed, Yvonne Gladbach, Steffen Möller, Sarah Fischer, Mathias Ernst, Stephan Struckmann, Alexander Storch, Georg Fuellen

**Affiliations:** 1 Institute for Biostatistics and Informatics in Medicine and Ageing Research, Rostock University Medical Center, Rostock, Germany; 2 Department of Neurology, University of Rostock, Rostock, Germany

## Abstract

The volume of molecular observations on human diseases in public databases is continuously increasing at accelerating rates. A bottleneck is their computational integration into a coherent description, from which researchers may derive new well-founded hypotheses. Also, the need to integrate data from different technologies (genetics, coding and regulatory RNA, proteomics) emerged in order to identify biomarkers for early diagnosis and prognosis of complex diseases and therefore facilitating the development of novel treatment approaches. We propose here a workflow for the integrative transcriptomic description of the molecular pathology in Parkinsons’s Disease (PD), including suggestions of compounds normalizing disease-induced transcriptional changes as a paradigmatic example. We integrated gene expression profiles, miRNA signatures, and publicly available regulatory databases to specify a partial model of the molecular pathophysiology of PD. Six genetic driver elements (2 genes and 4 miRNAs) and several functional network modules that are associated with PD were identified. Functional modules were assessed for their statistical significance, cellular functional homogeneity, literature evidence, and normalizing small molecules. In summary, our workflow for the joint regulatory analysis of coding and non-coding RNA, has the potential to yield clinically as well as biologically relevant information, as demonstrated here on PD data.

## Introduction

Parkinson’s disease (PD) is a progressive neurodegenerative disorder that affects several regions of the brain, particularly the substania nigra (SN) dopamine neurons controlling balance and movements^1,2^. It is considered to be the second most frequent neurodegenerative disorder after Alzheimer’s disease with a prevalence of approximately 180 per 100,000 inhabitants leading to ≈1,260,000 affected individuals in the European population^3,4^. Since both the incidence and prevalence of PD is increasing with age as for all neurodegenerative diseases^3^, the frequency of PD is continuously increasing in ageing societies, and thus PD and other neurodegenerative diseases become more and more important for their health care systems. The current treatment strategies only include symptomatic therapies using dopamine replacement by levodopa or dopamine agonist administration, antiglutamatergic or anticholinergic strategies or deep brain stimulation, but no causative therapeutic approaches have been identified. To identify biomarkers for early diagnosis and prognosis and to develop innovative causative treatments, detailed knowledge of the genetic causes and major determinants and regulators driving the underlying neurodegenerative process is an obligate prerequisite. However, due to the complex and heterogenic nature of the disease^5–7^, many of the cellular pathways and molecular regulators underlying PD pathology have not been unraveled so far.

Among the molecular regulators, transcription factors (TFs) and microRNAs (miRNAs) are key players for regulating gene expression^8^. Together they play crucial roles in regulating cellular processes^9^, and their malfunction can cause genetic disorders as well as complex diseases such as PD^2,10,11^. More specifically, TFs and miRNAs frequently form Feed Forward Loops (FFLs) and other network motifs to regulate cellular transcription in a connective manner^10,12,13^. Therefore, utilizing the combined regulatory information on TFs/genes and miRNAs as well as their target genes can shed light on key driver genes/TFs and miRNAs in human diseases and, in turn, suggest novel therapeutic strategies^14^.

Recent advances in molecular genetics revealed several factors implicated in the etiology of PD, such as inherited genetic predispositions and environmental factors that lead to acquired alterations^15,16^. For instance, it was demonstrated that SN neurons of PD patients show aberrant gene expression patterns that affect critical signaling pathways^2,17,18^. Moreover, miRNA expression analysis of human SN neurons showed distinct profiles that are dysregulated in PD patients^2,19,20^ and hence suggested critical functional roles of miRNAs in SN neurons as well as in PD pathogenesis as proposed in^21,22^.

Despite these important findings based on separate analyses of gene and miRNA expression, there is a lack of studies of the associated miRNA-mRNA interactome as well as of their co-regulation mechanisms, and of their collaborative functional roles in the underlying pathogenesis of PD. For instance, Chandrasekaran and co-workers 2013 analyzed several microarray gene expression datasets and biomolecular networks to identify novel genes and miRNAs of relevance to PD^23^. Also Dong *et al*. 2016 predicted ten potential therapeutic targets by studying the overlap between the differentially expressed genes and the target genes of the differentially expressed miRNAs in PD samples^24^. In the context of neurodegenerative diseases in general, we recently established a consensus-based strategy to integrate miRNA and gene expression data in multiple sclerosis to unravel the unknown cellular roles of miRNAs in this neuroimmune disease^25^.

As of today, the community has not yet agreed on best practices for such a joint analysis. A particular difficulty lies in the identification of gene-regulatory players and processes, especially since the control units of miRNA expression levels and the miRNAs they control were often found on different chromosomes^26^. This is a distinct difference with respect to coding RNA, which more frequently features cis-acting control^27^. An effective joint analysis will hence require the integration of a series of tools with tailored parameters for the interpretation of each dataset to reveal the regulatory mechanisms of disease pathogenesis.

**Figure 1:**
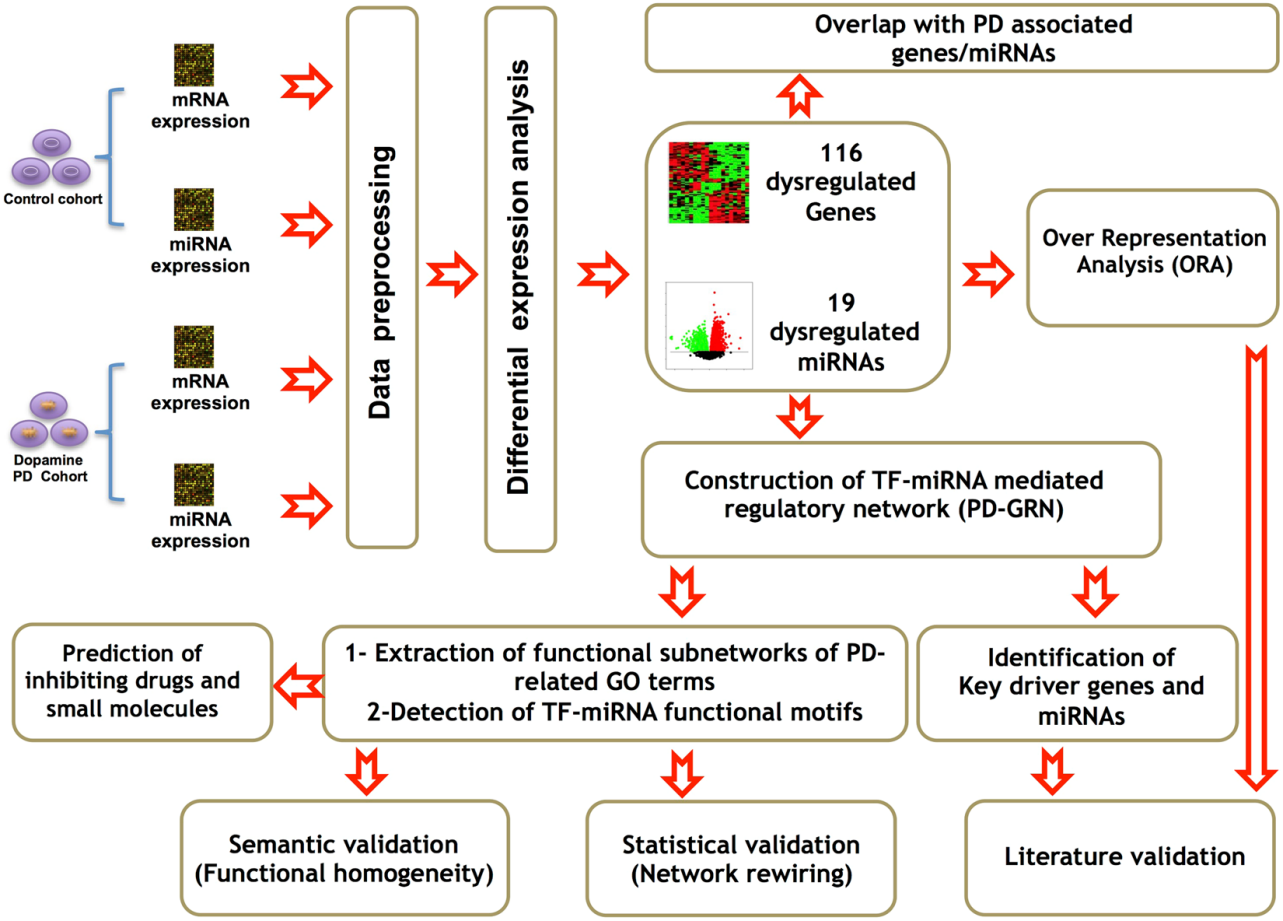
A schematic diagram for the integrative transcriptomic workflow. The sketch describes data processing and integration of two different transcriptomic datasets to detect major determinants and functional modules controlling PD.

To this end, we propose a comprehensive workflow for integrating miRNA expression profiles, gene expression profiles, and publicly available regulatory databases, in order to gain a deeper understanding of the co-regulation mechanisms and the collaborative functional role of miRNAs and genes in driving disease processes (see Fig. 1). The present study applied the workflow to PD as a paradigmatic example and by doing this yielded 19 dysregulated miRNAs and 116 dysregulated genes that may contribute to the molecular alterations behind PD. Out of these dysregulated genes and miRNAs, 24 genes and 3 miRNAs were found to be well known biomarkers in PD. Furthermore, we constructed a combinatorial gene regulatory network and highlighted six central hub nodes (two dysregulated genes and four dysregulated miRNAs) that could act as PD drivers; further experimental research is warranted to confirm these findings. Next, we identified recurring network motifs (functional modules) of dysregulated genes/TFs and miRNAs and co-targeted genes and we validated the cooperative functional role of their elements in the regulatory activities during PD pathogenesis in terms of statistical significance and biological relevance. Finally, we characterized small compounds and neuroprotective drugs that may normalize the regulatory activity of these functional modules.

## Results and Discussion

### Workflow description

We developed and applied an integrative bioinformatics workflow to conduct an integrative analysis with the aim of identifying the major genetic drivers and co-operative functional network modules that describe the molecular pathology of the disease and hence suggest disease-modulating drug compounds (Fig. 1). The workflow starts out with the separate analyses of expression data of mRNAs and miRNAs, including tests for differential expression. The differential expression data are then subject to combinatorial network analysis for the identification of hub nodes (i.e. candidate mRNAs/miRNAs that may drive the disease) as well as important functional network modules. The descriptive transcriptomics part concludes with a listing of these candidate genes and functional modules in the context of the current insights of the studied disease. We extended the workflow for an automated selection of drugs from LINCS^28^ and the Connectivity Map (CMap)^29^ with predicted normalizing effects on the dysregulated functional modules which may drive the disease. Formerly, we successfully confirmed the usefulness of multiple components of the workflow by applying it to a large number of breast cancer samples derived by different expression technologies to unravel the complex regulatory architecture of breast invasive carcinoma^13^. Apart from disease pathogenesis but in the context of cellular processes, we utilized the workflow in part to explain how imprinted genes contribute to cellular differentiation processes and to developmental stages of hematopoiesis^30^. Here we demonstrate the efficacy of the workflow in describing the molecular pathology of Parkinson’s disease, a disease with a limited number of biological samples.

### Differential analysis and enrichment analysis

**Figure 2:**
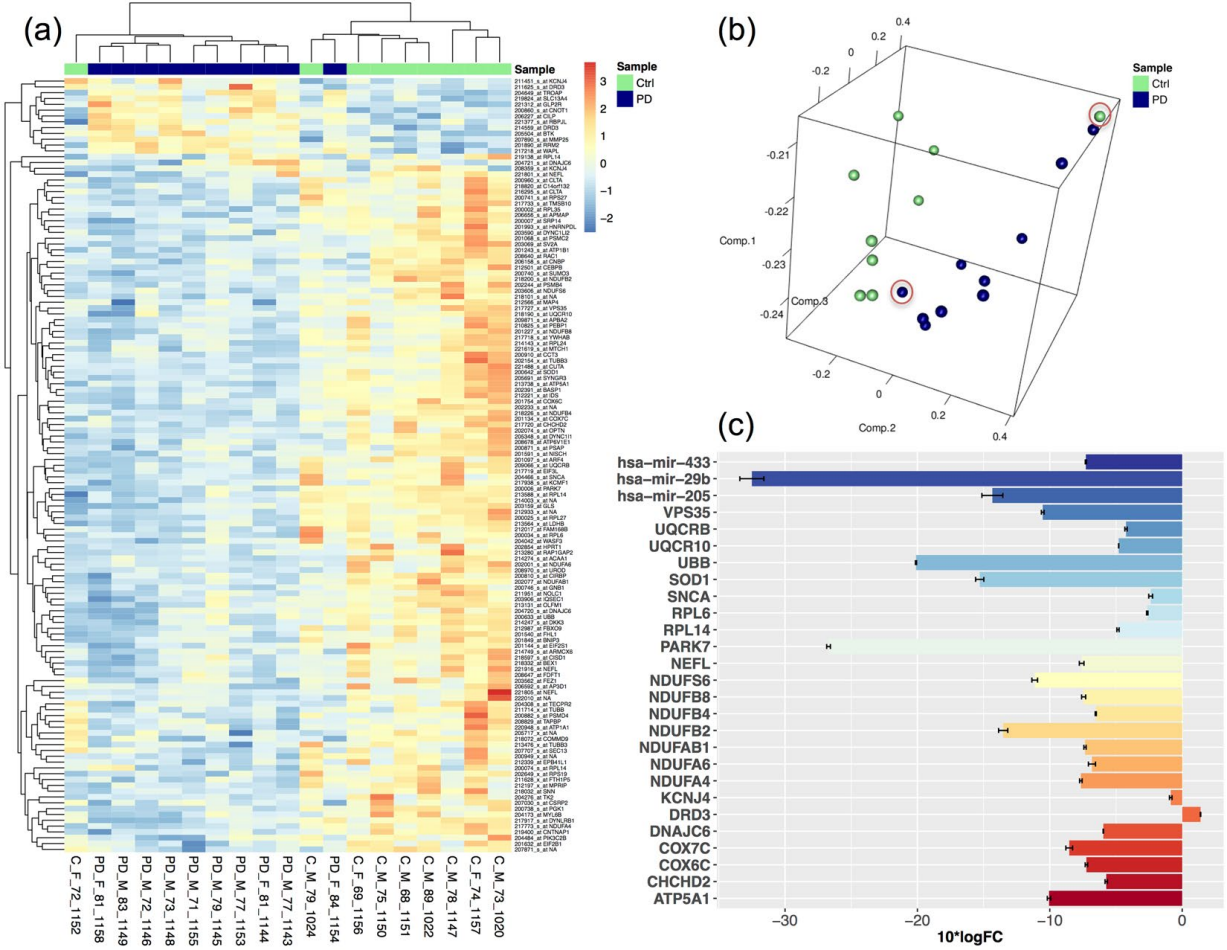
Differential analysis of gene expression and sample clustering. (**a**) The heatmap of the expression patterns of the 116 identified dysregulated genes between the PD and the control cohorts. Blue spots represent down-regulation whereas red-yellow spots denote up-regulation patterns. The dendrograms on the upper and left sides show the hierarchical clustering tree of genes or samples. (**b**) The PCA clustering for the normalized gene expression samples. The two highlighted PD and control samples are incorrectly clustered to the corresponding cohort, however they had almost no impact on the analysis results when we excluded them. (**c**) The Log fold change (LFC) of the 24 dysregulated genes and the 3 dysregulated miRNAs, which are known to be highly associated with PD progression and pathways. Each colour refers to a different gene or miRNA. The heatmap and PCA clustering for miRNA samples are shown in Figure S1.

We processed gene and miRNA expression datasets for 10 early-onset PD samples and 9 control samples of healthy tissues from previous PD studies (see Methods). These studies adopted separate gene or miRNA expression analyses and provided evidence for correlations of genes or miRNAs with signaling pathways relevant to PD pathogenesis^2,19^. Here we were concerned with the integrative analysis of both miRNA and gene expression datasets. PCA analysis of the normalized gene expression data clustered the analyzed samples into the corresponding groups (PD/Control) with only one exception per group (see Fig. 2). These two samples may be mislabeled. Nevertheless, they had only negligible impact on the results, as demonstrated when excluding them.

The differential expression analysis of the gene and miRNA expression data resulted in 116 dysregulated genes and 19 dysregulated miRNAs, respectively. Figure 2a shows the heatmaps for the relative expressions values of the dysregulated genes between the early-onset PD samples and the control samples. Interestingly, among these dysregulated genes and miRNAs, the genes: *PARK7*, *SNCA*, *VPS35*, *CHCHD2*, *UBB*, *NEFL*, *SOD1*, *KCNJ4*, *RPL14*, *RPL6*, *NDUFA4*, *NDUFB4*, *NDUFB8*, *NDUFA6*, *NDUFAB1*, *NDUFB2*, *COX7C*, *COX6C*, *NDUFS6*, *UQCR10*, *ATP5A1*, *UQCRB*, *DNAJC6*, *DRD3*, and the miRNAs: *hsa-mir-433*, *hsa-mir-205*, *hsa-mir-29b* were found to be strongly associated with PD pathways as annotated in DisGeNET^31^, OMIM^32^, HMDD^33^, or KEGG^34^ databases, and the list of genes is congruent with the list of genes described in^35^. All these genes and miRNAs were markedly downregulated in the PD samples except for the *DRD3* gene, which was upregulated, see Fig. 2c. The heatmap visualization of the dysregulated miRNAs and the PCA clustering for the miRNA samples are depicted in Figure S1.

The postulated functional roles of the dysregulated genes and miRNAs were backed up by inspecting the associated GO terms and KEGG pathways via overrepresentation analysis (ORA). We identified the most significant functional categories and processes that were enriched in the dysregulated miRNA and gene sets, demonstrating relevance to the etiology of PD, see Tables 1 and S1, respectively. Notably, neurological GO terms and pathways were among the top over-represented entries. For instance, the dysregulated genes were enriched with the functional terms GO:0006119 “*oxidative phosphorylation*” (*p* = *1*.*08E*^*−5*^), GO:0001963 “*synaptic transmission*, *dopaminergic*” (*p* = *0*.*001*), GO:0048699 “*Generation of neurons*” (p = 0.002), and the signaling pathways of each of Parkinson’s disease (*p* = *2*.*09E*^*−6*^), Huntington’s disease (*p* = *1*.*62E*^*−8*^), and Alzheimer disease (*p* = *4*.*22E*^*−8*^). Similarly, the dysregulated miRNAs were found to be enriched with cell death function (*p* = *0*.*005*), neurodegenerative diseases (*p* = *0*.*019*), and Parkinson’s disease (*p* = *0*.*04*), indicating  the cellular functional roles of the identified dysregulated genes and miRNAs in PD SA neurons.

**Table 1:** Enrichment of functional terms, diseases, and tissue specificity within the dysregulated miRNAs.

Category	Term	Count	adj p.value	miRNAs
Function	Epithelial-mesenchymal transition	4	0.0399	hsa-let-7b,hsa-mir-370,hsa-mir-382,hsa-mir-205
Function	Cell death	6	0.0059	hsa-let-7b,hsa-mir-130b,hsa-mir-129-5p,hsa-mir-205,hsa-mir-885-5p,hsa-mir-212
Disease	Heart Failure	9	0.0027	hsa-let-7b,hsa-mir-520d-5p,hsa-mir-129-5p,hsa-mir-139-3p,hsa-mir-382,hsa-mir-205,hsa-mir-130b,hsa-mir-212,hsa-mir-29b
Disease	Leukemia Myeloid Acute	5	0.0064	hsa-let-7b,hsa-mir-323-3p,hsa-mir-382,hsa-mir-29b,hsa-mir-370
Disease	Neurodegenerative Diseases	2	0.0199	hsa-let-7b,hsa-mir-139-3p
Disease	Parkinson’s Disease	2	0.0481	hsa-mir-433,hsa-mir-29b
Disease	Stomach Neoplasms	5	0.0297	hsa-mir-129-5p,hsa-mir-139-3p,hsa-mir-212,hsa-mir-433,hsa-mir-130b
TissueSpecific	Adrenal	2	0.0145	hsa-mir-370,hsa-mir-485-5p
TissueSpecific	Brain	4	0.0007	hsa-mir-323-3p,hsa-mir-383,hsa-mir-433,hsa-mir-129-5p

### Construction of TF-miRNA mediated regulatory network

**Figure 3:**
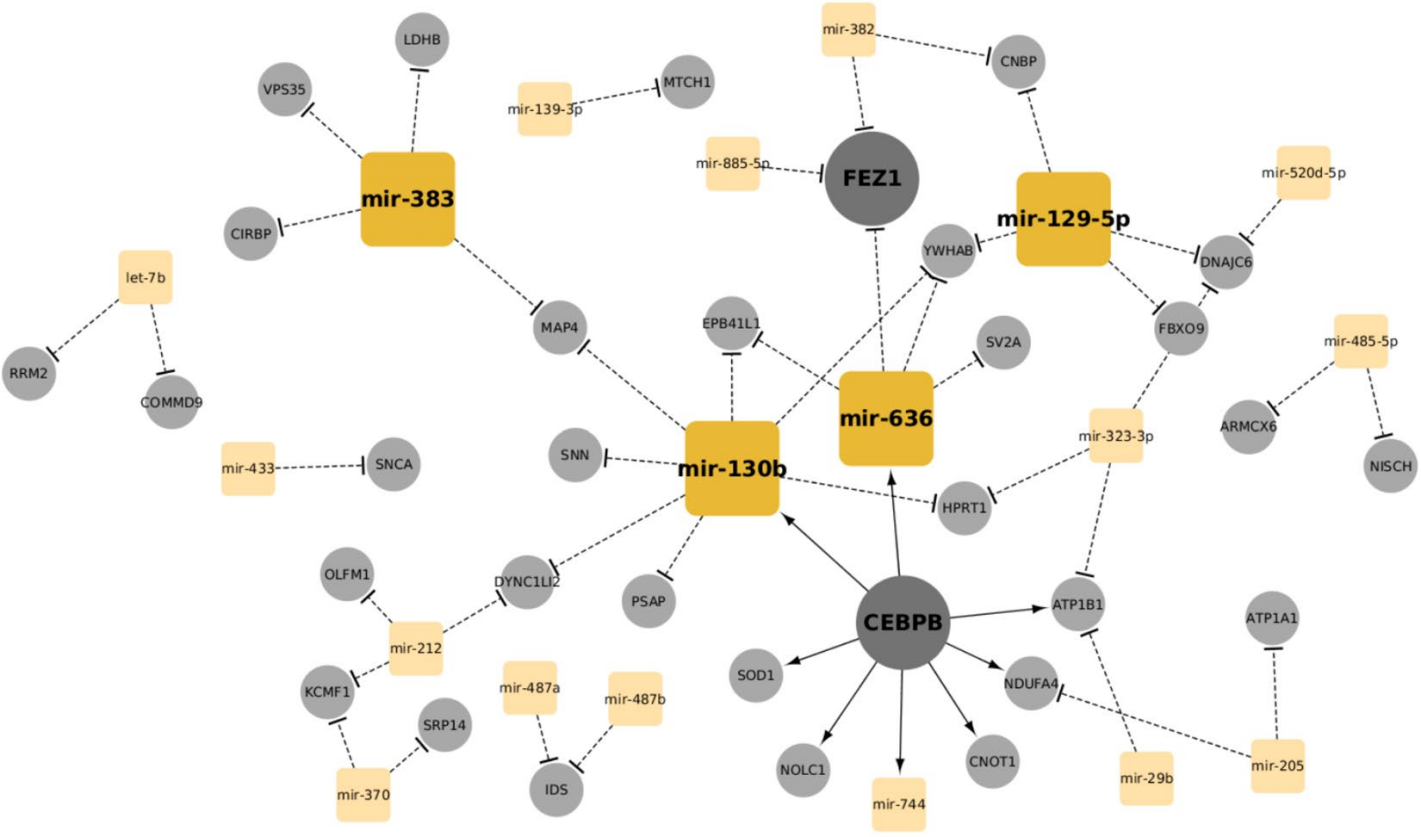
The PD gene regulatory network (PD-GRN) constructed from the dysregulated genes and miRNAs. Large nodes represent key driver genes and miRNAs. Square orange nodes denote the miRNAs, whereas the circular grey nodes represent genes. The network was visualized using the Cytoscape tool.

Next we constructed a GRN network (PD-GRN) that combines transcriptional and post-transcriptional regulatory interactions between the dysregulated genes and miRNAs (see Methods). The GRN network contains three different types of interactions: dysregulated gene/TF regulating a dysregulated gene (TF → mRNA), dysregulated miRNA regulating a dysregulated gene (miRNA → mRNA), and dysregulated gene/TF regulating a dysregulated miRNA (TF → miRNA), describing how miRNAs are an essential part of a complex regulation system in PD pathology. In order to characterize the central hub nodes that contribute a large amount of the overall regulation, we computed the node degree centrality parameters and ranked the nodes according to their degrees. We identified 4 central hub miRNAs (*hsa-mir-130b*, *hsa-mir-636*, *hsa-mir-383*, *hsa-mir-129-5p*) and 2 hub genes (*CEBPB*, and *FEZ1*) (Fig. 3). These hub nodes correspond to highly central nodes in the PD-GRN and play a critical role in maintaining the interactions between these genes and their neighborhood genes. Hence, they are candidates for master regulatory genes or for essential genetic drivers, and potential targets for new drugs and treatment of PD. Remarkably, the gene *CEBPB* was previously identified as a critical central hub node in a protein-protein interaction network related to neuron function activity in PD patients^36^. *CEBPB* is also early regulated by *CREB*^37^, which is one of the key transcription factors mediating adaptive responses of neurons to bioenergetics challenges^38^. Many studies have also reported the aberrant expression patterns of the *FEZ1* gene and its role in the regulation of the neuronal microenvironment during the progression of PD^39,40^. Also, expression disruption of the identified key miRNAs *hsa-mir-130b*, and *hsa-mir-636* has been connected to pathogenesis in neuropsychiatric and other neurodegenerative disorders^41–44^.

**Figure 4:**
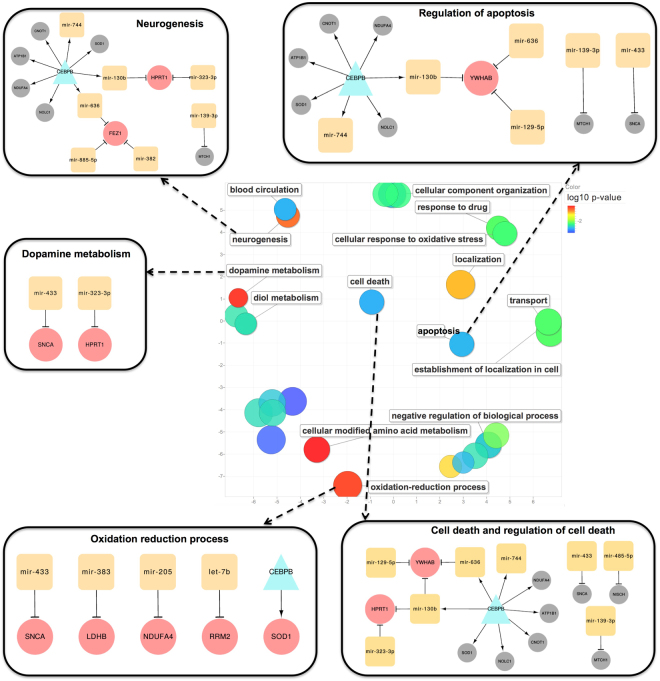
Enrichment analysis of the PD-GRN genes and visualization of five network modules corresponding to PD-related GO terms. Five GO terms are often affected in PD cases. The total list of the enriched GO terms is found in Table S2. The central scatter plot shows the visualization of the top enriched generic GO terms of the PD-GRN in a two dimensional space based on the GO semantic similarities. GO term node colours indicate the p-values for the enrichment of the GO terms. These generic GO terms represent implicitly their subterms, which are not visualized in the plot. The scatter plot was generated using the web tool REVIGO^79^. All five network modules include both miRNAs and genes. The main TF *CEBPB* is highlighted by a cyan triangle while the miRNAs are represented by orange squares. The genes co-targeted by TFs and/or miRNAs are depicted in larger pink circles. The regulated genes, regulated by a TF or by a miRNA, are coloured in grey. The network modules were visualized using the Cytoscape tool.

Subsequently, an enrichment analysis on all nodes of the PD-GRN network using DAVID was performed. We discovered a total of 64 enriched GO terms (adjusted p-value < 0.05) that are mostly neurology-related (see Table S2). Guided by the PD GO annotation project (a comprehensive resource providing GO annotation to PD-relevant human gene products)^45^, we found five important PD-related GO terms that are closely  related to PD, in our enrichment list (Fig. 4). We further inspected the association of miRNAs in the PD-GRN to these five GO terms in order to associate specific miRNAs to possible dysregulated pathways in PD. Therefore, for each of the five PD-related functional terms, we created a subnetwork module using the genes associated with these enriched terms as well as their neighboring nodes (Fig. 4). For example, the cell death subnetwork is created from the genes belonging to the enriched GO terms “*cell death*” and “*regulation of cell death*”, and their direct miRNA neighbors. There are eight miRNAs in this subnetwork and they regulate both the genes belonging to the enriched GO terms as well as other neighboring nodes. This functional subnetwork therefore provides us with dysregulated pathways concerning cell death in PD. From Fig. 4, we observe that *SOD1*, *CEBPB*, and *SCNA* are annotated with four out of the five GO terms, suggesting that these genes are apparently implicated in various dysregulated pathways in PD. These findings are also supported by recent studies showing the potential roles of *SCNA*^46,47^, *SOD1*^48^, and *CEBPB*^2,36^ in the neuronal loss in the PD brain and in PD development. Additionally, the miRNAs *mir-130*, *mir-636*, and *mir-744* are involved in subnetworks created from enriched GO terms corresponding to abnormal adult neurogenesis, apoptosis, and cell death. This hints at the role of these miRNAs in PD pathogenesis by modulating cell death as well as inhibiting the creation of new functional neuron cells.

### Identification of TF-miRNA co-regulatory motifs and statistical validation

**Figure 5:**
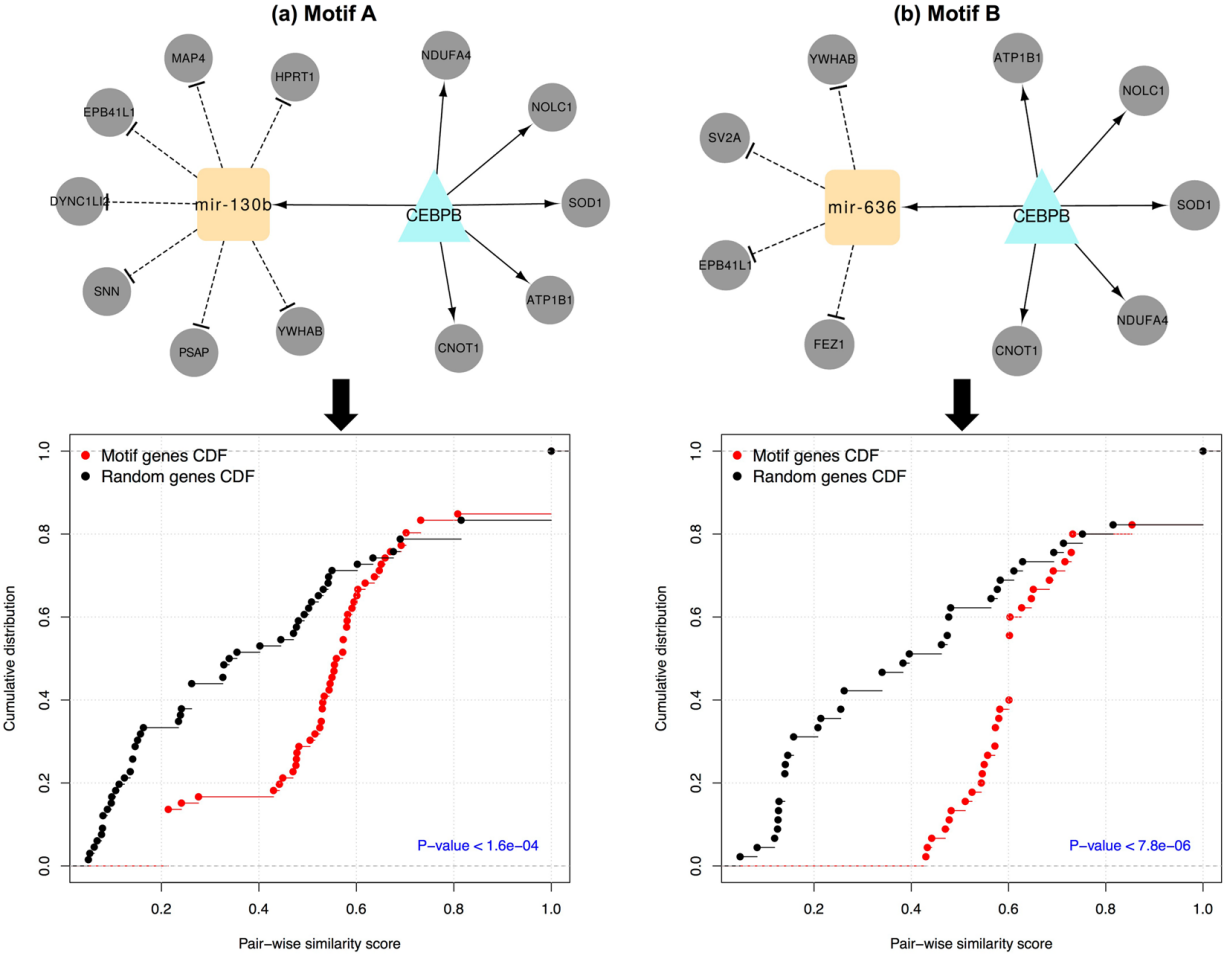
The reduced visualization of the detected motifs in the PD-GRN network. Motifs A and B and associated functional homogeneity plots depicting the cumulative distribution of GO functional semantic scores of gene pairs of co-regulated genes in the examined motif (red) versus randomly selected genes (black). The p-value was calculated using the Kolmogorov-Smirnov test. The network motifs were visualized using the Cytoscape tool.

Transcriptional gene regulatory networks often contain functional recurring patterns known as network co-regulatory motifs^49^ that control multiple features of normal cell function and that may trigger genetic disorders^50,51^. Here, our workflow checks for the presence of 3-node motifs involving the dysregulated genes and the dysregulated miRNAs in the PD-GRN network (see Methods). We also considered the motif types that were previously described in^52^. We unveiled a total of 11 cascaded-miRNA-mediated motifs comprising distinct combinations of a TF, a miRNA, a target gene, and co-regulated genes (Table S3). The statistical significance of the motifs was tested by comparing their counts in the network under investigation to their counts in randomized variants of these networks preserving the same node degrees *(p-value* = *0*.*03)*. Interestingly, the 11 motifs included mainly the TF *CEBPB* (see above) as a main regulator and varied between the two miRNAs *hsa-mir-130b* and *hsa-mir-636* as well as various target genes (Table S3). Therefore, we reduced the 11 motifs to two major motifs (Motif A and Motif B) as shown in Fig. 5.

Specifically, motif A, which involves the TF *CEBPB*, the miRNA *hsa-mir-130* and various target genes, is an example of how co-regulatory network motifs may help to better understand the pathogenicity of PD. Intriguingly, the *CEBPB* gene is involved in PD-related regulatory interactions via binding to the proximal promoter of the ATXN gene (which is the spinocerebellar Ataxia protein associated with the phenotypic variability of neurodegenerative diseases^53^) and thereby up-regulating its expression in neuroblastoma cells^54^. The ATXN *transcript* did not show dysregulation. However, Lee and colleagues 2008 demonstrated that *hsa-mir-130* regulates ATXN *protein* levels in human cells and its inhibition enhances the cytotoxicity caused by the ATXN *protein*^55,56^. This sheds light on the potential collaborative role between the hub gene *CEBPB* and the hub miRNA *hsa-mir-130* and their co-regulated genes/miRNAs in regulating ATXN. This strongly confirms our findings and implies that the identified motifs represent functional network modules that might be therapeutic targets for PD.

### Semantic assessment: functional homogeneity within the motif nodes

Next, the biological evidence for the two co-regulatory motifs A and B is evaluated in more depth to better assess their cooperative functional roles during the etiology of early-onset PD. We measured the functional homogeneity within each motif by calculating the functional similarity scores between all gene pairs and comparing the resulting distribution to the similarity score distribution of randomly selected gene pairs from the network (p-values < 1.6E-4, Kolmogorov-Smirnov test), see Fig. 5. Intriguingly, the motif genes have significantly more cellular functional homogeneity than randomly selected ones. Therefore, these motifs could highlight new insights into TF-miRNA network modules in PD development, by demonstrating the cooperative functional role between the TFs/genes and their potential miRNA partners, yielding a better understanding of the dysregulation mechanisms involving PD pathways.

Finally, we merged the two main motifs A and B to obtain a consolidated functional network module with their co-regulated genes that are enriched in the PD-GRN, see Figure S2.

### Identification of normalizing small molecules and drugs

Our workflow concludes by proposing small molecule interventions that possibly normalize the expression signatures of the merged functional module (Figure S2) using the library of integrated network-based cellular signatures (LINCS) L1000 data set^28,57^, by identifying (based on the complete extrapolated set of genes, not the landmark genes only) small molecules causing transcriptional changes inversely correlating with our expression signatures. We used an optimized PD signature (the merged functional network module) instead of using all differentially expressed genes or a whole genome expression profile as a signature for the LINCS query with the aim of obtaining superior performance for compound prioritization and assessing the biological relevance of the identified functional modules. Interestingly, LINCS identified known neuroprotective agents (e.g., Staurosporine and Brivanib) and signaling pathway inhibitors (e.g., GDC-0068 and Torin-2), which work on the central nervous system to prevent neuron degradation^58^ and to delay progression of PD^59^ and Alzheimer^60^ in animal models, see Table S4. This supports the hypothesis that the identified functional network modules are relevant for PD, and may open up new avenues for therapy.

Subsequent to the LINCS methodology, observed changes in gene expression were compared against the drug effects described by the CMap^29^ to identify the drugs that may reverse the whole genome expression profile of PD cases. For each of the 3203 gene expression profiles in the CMap, we generated Rank-rank hypergeometric overlap (RRHO) maps^61^, showing the extent and pattern of its global similarity to our inverted PD gene expression profile, see Figure S3. We were interested especially in CMap profiles that feature upregulation of genes that are downregulated in the PD profile (i.e. at the top of the list in the inverted PD profile). In a RRHO map such behavior would materialize as a region of high intensity (reflecting low overlap p-values; red color) in the lower left corner of the map, with lower intensities elsewhere. Thus, we computed for each of the 3203 RRHO maps the degree of similarity (distance) to an artificially constructed reference map showing that intensity distribution and ranked the list of RRHO maps accordingly. Figure S3 depicts a panel of 16 maps from the top of that list, i.e. drugs from the CMap whose upregulated genes show considerable overlap with genes downregulated in PD. Among those are *hesperetin* and *valproic acid* which were found to have neuroprotective effects by attenuating behavioral abnormality in hemiparkinsonian rats^62^, and by reversing the alpha-synuclein alterations in a rotenone rat model of PD^63^, respectively.

### Comparison to similar workflows/approaches

Several approaches have been implemented to facilitate the integrative analysis of gene and miRNA expression profiles. For instance, the MMIA web tool integrates miRNA and mRNA expression data using straightforward inverse correlation between the mis-regulated genes and miRNAs as well as gene set enrichment analysis to characterize diseases and pathways related to miRNAs^64^. The MAGIA^65^ pipeline provides a higher diversity by allowing the combination of miRNA-target gene predictions for either matched or un-matched miRNA–gene expression profiles using different relatedness measures to end up with a regulatory network for associated phenotypes. Also miRTrail^66^ performs ORA and Gene Set Enrichment analyses of interactions of genes and miRNAs based on expression profiles. However, it explores only miRNA → gene interactions. DisTMGneT^67^ was developed for obtaining a cancer-specific network based on expression profiles of dysregulated genes and miRNAs. However, it is limited to a predefined set of miRNAs and genes as well as cancer. Compared to the aforementioned approaches, our workflow has distinctive features of downstream analysis such as identification of driver genes/miRNAs, detecting effective functional network modules, small molecule predictions, and a variety of validation methods. Table S5 summarizes the comparison between our approach and the aforementioned approaches/tools.

## Conclusions

We propose a workflow for the joint analysis of coding and non-coding RNA taken from the same samples. It has the potential to yield clinically as well as biologically relevant information, as demonstrated here on PD data, with a very limited number of samples and previously on breast cancer data with a large number of samples. Furthermore, it offers descriptions of distinct molecular processes that are associated with the disease, provides consistent enrichments of Gene Ontology terms and of disease pathways, and identifies cooperative functional modules. For each functional module a network of interacting coding and non-coding transcripts is offered. The resulting data are also subjected to a comparison with drug effect databases to give researchers the opportunity to identify hypotheses for curative effectors. We consider that this workflow nicely links clinical studies (from which the expression data may be derived) back to preclinical research. The presented workflow represents an effective model for the integrative analysis of multiple molecular datasets from different experimental assays. Further work to increase the modularity of this workflow is warranted to ease the exchange of tools for the generation of networks and to foster reusability.

## Methods

### Material collections and experimental procedures

Genetic material collection, DNA preparation, and microarray experimental procedures for gene and miRNA expression in midbrain dopamine neurons were previously described in the two reference publications for the data we utilized^2,19^. These authors obtained frozen tissue blocks from the Harvard Brain Tissue Resource Center, containing SN from 9 control subjects and 10 idiopathic PD samples, which were cut using a Microm HM 560 CryoStar cryostat (8 μm), mounted on LEICA Frame Slides with a PET-membrane (1.4 μm), dehydrated, subjected for RNA extraction, followed by hybridization to the HU-133A arrays (Affymetrix, Santa Clara, CA). From these 19 samples, 8 control and 8 PD samples were also used for miRNA profiling using the Human MicroRNA TaqMan qRT-PCR Arrays A v1 or 2.0 (Life Technologies, Foster City, CA, USA).

### Pre-processing and differential analysis

The raw expression datasets were normalized using quantile normalization and log2 transformed. The differential expression analysis was performed as previously described^68^ using three methods: (1) ANOVA test^69^, (2) moderated t-test^70^, (3) area under the curve of the receiver operator characteristics (AUC ROC)^70^. P-values were adjusted using the Benjamini-Hochberg^71^ procedure to limit the false discovery rate to 5%. Genes/miRNAs that were classified as differentially expressed by at least two of the three methods were included in the list of differentially expressed genes/miRNAs. Raw and preprocessed data are provided as a supplementary file.

### Construction of the PD-GRN network and the co-regulatory motifs

The regulatory interactions between the differentially expressed (DE) genes and the DE miRNAs, which were identified in this work, were collected from the TFmiR regulatory databases^13^. We considered all interactions that are supported by experimental and/or by predicted evidence in this analysis. Driver genes/miRNAs (hub nodes) were identified by determining the highly central nodes in the constructed PD-GRN network. For this, we calculated the degree centrality measure for the PD-GRN network using the R package igraph^72^ and we selected the top 10% (of all genes and miRNAs) only to foster the consistency in their association to GO biological processes^73^. Functional network modules (three-node TF-miRNA co-regulatory network motifs consisting of a miRNA, a TF, and a joint target gene) were characterized using the computational procedure described in the TFmiR web server publication^13^. Cytoscape V3.3^74^ was used to visualize the PD-GRN network and the identified network motifs.

### Assessment of driver genes and functional network modules

The following 4-step procedures were used to assess and validate our results:

#### Significance of the identified network motifs (Statistical validation)

To assess the significance of each motif type, we used the same procedure that we previously developed in^13^. Briefly, we compared the motif occurrences in the real network to their occurrences in randomized ensembles of these networks with preserved node degrees (number of permutations = 100). Only motifs having p-value < 0.05 were considered for further analysis.

#### Functional homogeneity within the motif genes (Semantic validation)

We used the GoSemSim R package^75^ to estimate semantic similarity scores according to the Gene Ontology (GO) annotations. Statistical significance was performed by comparing the similarity scores of the motif genes to the similarity scores of randomly selected genes (with the same number of the motif genes). The permutation procedure was repeated 100 times. Then, the Kolmogorov-Smirnov test was adopted to check whether the similarity scores of motif gene pairs were statistically higher than the scores of randomly selected pairs.

#### Over-representation analysis for genes and miRNAs

Over-representation analysis (ORA) of the miRNA sets was performed using TAM web service^76^ which identifies the functional classes and disease terms that are enriched in a miRNA set. For the gene sets, the DAVID^77^ tool was utilized to identify the GO terms which are annotated to at least two genes and are statistically overrepresented in the DE genes as previously shown in reference^78^. For both genes and miRNA enrichment analysis, Fisher’s Exact test was performed followed by the Benjamini–Hochberg^71^ adjustment *for controlling the false discovery rate (FDR)*, with a cutoff value of 0.05.

#### Identifying inhibiting small molecules and drugs

- *For each functional module expression profile*
  We downloaded the complete version of the library of integrated network-based cellular signatures (LINCS) L1000 data set^28,57^ on level 3 from the GEO repository (https://www.ncbi.nlm.nih.gov/geo/query/acc.cgi?acc=GSE70138) and computed the log2 fold change (LFC) of all L1000 genes. The PD-related LFC of the genes involved in the functional network modules was subtracted from the same genes in the processed LINCS data and then summed across all genes in the module to end up with a representative score for each drug. Low-scoring drugs feature a maximum desired expression change on the entire gene set of the functional network module. The drug scores are sorted ascendingly and the first ten drugs were considered to provide biologists/clinicians with a limited but potentially worthwhile set of drug predictions.
- *For the whole genome PD expression profile*
  The genes in the PD gene expression profile were ranked based on the LFC, from the most downregulated gene to the most upregulated one (inverted profile). We also ranked the genes in each of the 3203 drug gene expression profiles from the ConnectivityMap (CMap)^29^ based on the log2 fold change from the most upregulated gene to the most downregulated one. For every pair of a PD profile and a CMap profile we computed a rank-rank hypergeometric overlap (RRHO) map^61^. A desirable match between the PD profile and a given CMap profile would result in a RRHO map with a characteristic intensity distribution. We constructed an artificial map depicting such an intensity distribution as a positive reference and compared the list of RRHO maps with it. All maps were normalized, serialized into a vector and column-wise assembled into a matrix. We performed Principal Component Analysis (PCA) using the rows of that matrix as features and determined the number of principal components (PCs) needed to account for 90% of the total variance. Using those PCs, we calculated a weighted Euclidean distance for each map to the reference map, with the weight reflecting the amount of explained variance for each PC. Finally, we ranked the RRHO maps by their distance to the reference map.

### Workflow reusability

This integrative workflow can be classified into two main software modules:

1. the differential analysis, concerning the identification of the differentially expressed genes/miRNAs. This module was developed as an R script and can be downloaded from the Bitbucket repository (https://hamed20@bitbucket.org/hamed20/differential-analysis.git). The outputs are tab-delimited files of differentially expressed genes/miRNAs, which can be used as input to the next module.
2. the combinatorial analysis, including the other workflow components. This analysis is accessible using our public webservice TFmiR: http://service.bioinformatik.uni-saarland.de/tfmir. The user guides are included in the corresponding software modules.

### Availability Data and Materials

Raw and pre-processed data are available as supplementary.

## Electronic supplementary material

Supplementary
