# Supplementary material for: A workflow for the integrative transcriptomic description of molecular pathology and the suggestion of normalizing compounds, exemplified by Parkinson’s disease
Source: Sci Rep. 2018 May 21;8:7937. doi: 10.1038/s41598-018-25754-5 (PMC5962550; doi:10.1038/s41598-018-25754-5)

Supplementary Information

A workflow for the integrative transcriptomic description of molecular pathology and the suggestion of normalizing compounds, exemplified by Parkinson's disease

Mohamed Hamed[1], Yvonne Gladbach[1], Steffen Möller[1], Sarah Fischer[1], Mathias Ernst[1], Stephan Struckmann[1] , Alexander Storch[2], Georg Fuellen[1§]

[1]Institute for Biostatistics and Informatics in Medicine and Ageing Research, Rostock University Medical Center, Rostock, Germany.

[2]Department of Neurology, University of Rostock, Rostock, Germany.

Supplementary Tables

Table S1 – Enrichment of the 116 dysregulated genes with GO terms and KEGG pathways. The table lists the top 30 significant GO terms and the entire list of the enriched pathways.

Category	Term	Count	Adj p-value
GOTERM_BP	GO:0006119~oxidative phosphorylation	12	1,08E-05
GOTERM_BP	GO:0042773~ATP synthesis coupled electron transport	10	2,14E-06
GOTERM_BP	GO:0042775~mitochondrial ATP synthesis coupled electron transport	10	2,14E-06
GOTERM_BP	GO:0022904~respiratory electron transport chain	10	7,43E-05
GOTERM_BP	GO:0006091~generation of precursor metabolites and energy	17	7,91E-05
GOTERM_BP	GO:0045333~cellular respiration	10	3,17E-07
GOTERM_BP	GO:0022900~electron transport chain	10	1,30E-09
GOTERM_BP	GO:0006120~mitochondrial electron transport, NADH to ubiquinone	7	5,21E-08
GOTERM_BP	GO:0015980~energy derivation by oxidation of organic compounds	10	9,53E-08
GOTERM_BP	GO:0006810~transport	39	7,21E-09
GOTERM_BP	GO:0006414~translational elongation	8	8,24E-09
GOTERM_BP	GO:0051234~establishment of localization	39	9,23E-09
GOTERM_BP	GO:0051179~localization	41	2,52E-11
GOTERM_BP	GO:0032269~negative regulation of cellular protein metabolic process	9	4,73E-10
GOTERM_BP	GO:0051248~negative regulation of protein metabolic process	9	6,19E-10
GOTERM_BP	GO:0006412~translation	11	1,38E-11
GOTERM_BP	GO:0016310~phosphorylation	17	1,89E-12
GOTERM_BP	GO:0031400~negative regulation of protein modification process	7	2,20E-11
GOTERM_BP	GO:0051258~protein polymerization	5	4,24E-12
GOTERM_BP	GO:0055114~oxidation reduction	14	6,80E-11
GOTERM_BP	GO:0070271~protein complex biogenesis	12	0.001
GOTERM_BP	GO:0006461~protein complex assembly	12	0.001
GOTERM_BP	GO:0030182~neuron differentiation	11	0.0012
GOTERM_BP	GO:0022008~neurogenesis	13	0.00129
GOTERM_BP	GO:0006796~phosphate metabolic process	17	0.0016
GOTERM_BP	GO:0006793~phosphorus metabolic process	17	0.00156
GOTERM_BP	GO:0001963~synaptic transmission, dopaminergic	3	0.0018
GOTERM_BP	GO:0048699~generation of neurons	12	0.0023
GOTERM_BP	GO:0046907~intracellular transport	13	0.0027
GOTERM_BP	GO:0065003~macromolecular complex assembly	13	0.003
KEGG_PATHWAY	hsa05012: Parkinson's disease	15	2.09E-06
KEGG_PATHWAY	hsa00190: Oxidative phosphorylation	13	3.41E-08
KEGG_PATHWAY	hsa05016: Huntington's disease	14	1.62E-08
KEGG_PATHWAY	hsa05010: Alzheimer's disease	13	4.22E-08
KEGG_PATHWAY	hsa03010: Ribosome	7	6.02E-11
KEGG_PATHWAY	hsa04260: Cardiac muscle contraction	6	0.0025

Table S2 – GO enrichment analysis of PD-GRN genes.

Term	Count	Adj p-value
GO:0006753~nucleoside phosphate metabolic process	5	0.003
GO:0009117~nucleotide metabolic process	5	0.003
GO:0055086~nucleobase, nucleoside and nucleotide metabolic process	5	0.003
GO:0044271~nitrogen compound biosynthetic process	5	0.004
GO:0051259~protein oligomerization	4	0.005
GO:0009165~nucleotide biosynthetic process	4	0.006
GO:0016043~cellular component organization	12	0.006
GO:0034654~nucleobase, nucleoside, nucleotide and nucleic acid biosynthetic process	4	0.006
GO:0034404~nucleobase, nucleoside and nucleotide biosynthetic process	4	0.006
GO:0006810~transport	12	0.009
GO:0042493~response to drug	4	0.009
GO:0051234~establishment of localization	12	0.009
GO:0006066~alcohol metabolic process	5	0.01
GO:0030182~neuron differentiation	5	0.01
GO:0010894~negative regulation of steroid biosynthetic process	2	0.016
GO:0045939~negative regulation of steroid metabolic process	2	0.016
GO:0008217~regulation of blood pressure	3	0.017
GO:0019216~regulation of lipid metabolic process	3	0.021
GO:0051179~localization	12	0.023
GO:0044057~regulation of system process	4	0.023
GO:0048699~generation of neurons	5	0.023
GO:0048666~neuron development	4	0.029
GO:0051055~negative regulation of lipid biosynthetic process	2	0.029
GO:0022008~neurogenesis	5	0.03
GO:0006164~purine nucleotide biosynthetic process	3	0.035
GO:0055114~oxidation reduction	5	0.036
GO:0016044~membrane organization	4	0.039
GO:0042417~dopamine metabolic process	2	0.039
GO:0048523~negative regulation of cellular process	8	0.039
GO:0006575~cellular amino acid derivative metabolic process	3	0.043
GO:0050810~regulation of steroid biosynthetic process	2	0.048
GO:0032269~negative regulation of cellular protein metabolic process	3	0.049
GO:0008219~cell death	5	0.005
GO:0003013~circulatory system process	3	0.005
GO:0006163~purine nucleotide metabolic process	3	0.005
GO:0008015~blood circulation	3	0.005
GO:0043065~positive regulation of apoptosis	4	0.005
GO:0051248~negative regulation of protein metabolic process	3	0.005
GO:0016265~death	5	0.005
GO:0043068~positive regulation of programmed cell death	4	0.005
GO:0010942~positive regulation of cell death	4	0.005

GO:0045833~negative regulation of lipid metabolic process	2	0.005
GO:0048519~negative regulation of biological process	8	0.006
GO:0007399~nervous system development	6	0.006
GO:0006916~anti-apoptosis	3	0.006
GO:0048667~cell morphogenesis involved in neuron differentiation	3	0.006
GO:0009712~catechol metabolic process	2	0.007
GO:0006584~catecholamine metabolic process	2	0.007
GO:0034311~diol metabolic process	2	0.007
GO:0009892~negative regulation of metabolic process	5	0.007
GO:0018958~phenol metabolic process	2	0.007
GO:0019218~regulation of steroid metabolic process	2	0.007
GO:0042981~regulation of apoptosis	5	0.007
GO:0043067~regulation of programmed cell death	5	0.007
GO:0010941~regulation of cell death	5	0.008
GO:0006461~protein complex assembly	4	0.008
GO:0070271~protein complex biogenesis	4	0.008
GO:0034599~cellular response to oxidative stress	2	0.008
GO:0051240~positive regulation of multicellular organismal process	3	0.008
GO:0000904~cell morphogenesis involved in differentiation	3	0.008
GO:0051649~establishment of localization in cell	5	0.009
GO:0046890~regulation of lipid biosynthetic process	2	0.009
GO:0043524~negative regulation of neuron apoptosis	2	0.01
GO:0048518~positive regulation of biological process	8	0.01

Table S3 – The list of TF-miRNA co-regulatory motifs identified in the PD-GRN network. *P-values* were computed for motif types (not for each individual motif) by comparing their count in the real network (PD-GRN) to their counts in randomized variants of these networks preserving the same node degrees. All identified motifs were belonging to one motif type (cascaded-miRNAs-mediated, which includes a TF regulation of the expression of a miRNA that in turns represses a target gene) with a corrected p-value 0.03. The BH method was used for multiple test correction.

Motif ID	Motif Type	TF	miRNA	Target	Co-regulated targets
1	CASCADED-MIRNA-MEDIATED	CEBPB	hsa-mir-130b	MAP4	NDUFA4, CNOT1, ATP1B1, SOD1, NOLC1, SNN, DYNC1LI2, EPB41L1, YWHAB, PSAP, HPRT1
2	CASCADED-MIRNA-MEDIATED	CEBPB	hsa-mir-130b	YWHAB	NDUFA4, CNOT1, ATP1B1, SOD1, NOLC1, SNN, DYNC1LI2, EPB41L1, PSAP, MAP4, HPRT1
3	CASCADED-MIRNA-MEDIATED	CEBPB	hsa-mir-130b	DYNC1LI2	NDUFA4, CNOT1, ATP1B1, SOD1, NOLC1, SNN, EPB41L1, YWHAB, PSAP, MAP4, HPRT1
4	CASCADED-MIRNA-MEDIATED	CEBPB	hsa-mir-130b	HPRT1	NDUFA4, CNOT1, ATP1B1, SOD1, NOLC1, SNN, DYNC1LI2, EPB41L1, YWHAB, PSAP, MAP4
5	CASCADED-MIRNA-MEDIATED	CEBPB	hsa-mir-130b	EPB41L1	NDUFA4, CNOT1, ATP1B1, SOD1, NOLC1, SNN, DYNC1LI2, YWHAB, PSAP, MAP4, HPRT1
6	CASCADED-MIRNA-MEDIATED	CEBPB	hsa-mir-130b	SNN	NDUFA4, CNOT1, ATP1B1, SOD1, NOLC1, DYNC1LI2, EPB41L1, YWHAB, PSAP, MAP4, HPRT1
7	CASCADED-MIRNA-MEDIATED	CEBPB	hsa-mir-130b	PSAP	NDUFA4, CNOT1, ATP1B1, SOD1, NOLC1, SNN, DYNC1LI2, EPB41L1, YWHAB, MAP4, HPRT1
8	CASCADED-MIRNA-MEDIATED	CEBPB	hsa-mir-636	YWHAB	NDUFA4, CNOT1, ATP1B1, SOD1, NOLC1, EPB41L1, SV2A, FEZ1
9	CASCADED-MIRNA-MEDIATED	CEBPB	hsa-mir-636	SV2A	NDUFA4, CNOT1, ATP1B1, SOD1, NOLC1, EPB41L1, FEZ1, YWHAB
10	CASCADED-MIRNA-MEDIATED	CEBPB	hsa-mir-636	EPB41L1	NDUFA4, CNOT1, ATP1B1, SOD1, NOLC1, SV2A, FEZ1, YWHAB
11	CASCADED-MIRNA-MEDIATED	CEBPB	hsa-mir-636	FEZ1	NDUFA4, CNOT1, ATP1B1, SOD1, NOLC1, EPB41L1, SV2A, YWHAB

Table S4 – Top ten predicted LINCS small molecules that reverse the transcriptional activities of the merged functional module and that therefore may inhibit the underlying regulatory mechanisms of PD pathogenesis. Interestingly, most of them were found to be neuroprotective agents or signaling pathway inhibitors that are relevant to neurodegenerative diseases.

Ranking based on drug score (see methods)	**Small molecule**	**Concentration**	**Application time**	**Cell type**	**Clinical Relevance to neurological diseases supported by literature evidences**
1	GDC-0068	0.04um	24h	CD34	GDC-0068 is an inhibitor for AKT signaling pathway, which has a crucial role in PD prognosis (https://www.ncbi.nlm.nih.gov/pmc/articles/PMC4499569/)
2	Brivanib	1.11um	24h	MDAMB231	Brivanib is a novel VEGF-R2/bFGF-R signaling inhibitor, which is used for mediating signals by Alzheimer's β-amyloid peptide (http://onlinelibrary.wiley.com/doi/10.1111/j.1471-4159.2009.06426.x/full).
3	Erlotinib	3.33um	24h	MDAMB231	Erlotinib is an EGFR inhibitor that delays the disease progression of ALS Amyotrophic lateral sclerosis (ALS), a neurodegenerative disease due to neuron death. (http://journals.plos.org/plosone/article?id=10.1371/journal.pone.0062342)
4	JWE-035	0.12um	24h	HME1	JWE-035 targets the aurora kinase A protein, which is used for glioblastoma therapy (http://thejns.org/doi/pdf/10.3171/2010.3.PEDS10120).
5	Torin-2	0.37um	24h	MDAMB231	Torin-2 is a mTor inhibitor that prevents neuron cell death in substantia nigra neurons from PD model (https://www.dovepress.com/recent-insights-into-the-pathophysiology-of-mtor-pathway-dysregulation-peer-reviewed-fulltext-article-RRB), (http://www.jneurosci.org/content/26/39/9996.long)
6	BX-912	10um	24h	MDAMB231	BX-912 targets PDK1, A key player in the PI3K signaling pathway, which is strongly deregulated in PD cases (https://www.ncbi.nlm.nih.gov/pmc/articles/PMC4068290/)
7	PHA-665752	0.37um	24h	MDAMB231	This small molecule is an inhibitor for c-Met pathway, which has been implicated in neuroprotection, especially against Alzheimer and PD (https://www.ncbi.nlm.nih.gov/pmc/articles/PMC3829467/).
8	HY-11007	0.04um	24h	HME1	HY-11007 is known by a product name "GNF-2", which is a BCR- ABL inhibitor. These inhibitors were examined regarding their neuroprotective or therapeutic role in PD (http://www.mdsabstracts.org/abstract/bcr-abl-tyrosine-kinase-inhibitors-b-atki-and-parkinsons-disease-pd/).
9	Staurosporine	1um	6h	MCF7	Staurosporine induces neuron apoptosis and has protective effects against PD (https://www.ncbi.nlm.nih.gov/pubmed/16079129).
10	SB590885	1.11um	24h	MDAMB231	This small molecule targets BRAF, whose inhibition by SB590885 leads to enhanced CNS penetration and might be valuable in stroke treatment (https://www.ncbi.nlm.nih.gov/pubmed/18621524).

Table S5 – A comparison between our approach and similar approaches/tools

**Approach**<br><br>**/Tool [reference]**	**Input data**	**Performed Analysis/ highlights**	**Output**	**Accessibility**	**Integrated ontologies**<br><br>**/tools**
**Our approach**	-miRNA, and mRNA expression	-Differential analysis for genes/ miRNAs<br><br>-Construction of regulatory interactions between deregulated miRNAs and mRNAs<br><br>-Gene Regulatory network analysis<br>-Enrichment analysis of deregulated genes and miRNAs<br><br>-Identification of putative driver genes/miRNAs<br><br>-Detection of functional network modules (network motifs)<br><br>-Semantic and statistical validation of the identified functional modules<br><br>-Prediction of normalizing small compounds	-Gene Regulatory network<br><br>-Disease specific network<br><br>-Significant functional network modules (3-node network motifs)<br><br>-Driver genes/miRNAs<br><br>-Enriched GO terms and pathways for genes and miRNAs<br><br>-Drugs normalizing the disease–induced transcriptional effects	-In two parts: R script and web service	-TRED<br><br>-Transmir<br><br>-Tarbase<br><br>-miRtarbase<br><br>-starBase<br><br>-ChIPBase<br><br>-PmmR<br><br>-DAVID<br><br>-HMDD<br><br>-DisGeNET
**MMIA [61]**	-miRNA, and mRNA expression	-Gene set enrichment analysis<br><br>-miRNA target prediction<br><br>-Inverse correlation analysis between miRNA and mRNA expression<br><br>-Identification of dysregulated miRNAs<br><br>-Identification of TFBS of miRNA target genes	-Diseases associated with miRNAs<br><br>-TFBS in promoter regions of predicted targets<br><br>-Enriched GO terms and Pathways	-Web service	-TargetScan<br><br>-PicTar<br><br>-PITA

**MAGIA [62]**	-miRNA, and mRNA expression	-Gene regulatory network of miRNA and mRNA interactions<br>-miRNA target prediction<br>-Gene set enrichment analysis<br>-Utilization of different association measures between miRNA and mRNA expression	-Top 250 most probable functional miRNA-mRNA interactions<br>-Bipartite regulatory network<br>-Links to DAVID, PubFocus, EBIMed	-Web service	-PITA<br>-miRanda<br>-TargetScan<br>-RNAhybrid
**miRTrail [63]**	-miRNA expression	-Identification of deregulated miRNAs<br>-miRNA target enrichment analysis<br>-Gene set enrichment analysis and over-representation analysis (ORA)<br>-Network visualization using BINA and Cytoscape	-Contingency table<br>-Venn diagrams for miRNA target overlaps with studied disease<br>-ORA for the deregulated miRNA and their gene targets<br>-Common pathway analysis<br>-Network analysis with ORA of custom selected network modules	-Web service	-microCosm<br>-miRanda<br>-geneTrail<br>-NIH human disease<br>Datasets
**Dis TMGneT [64]**	-Predefined set(s) of cancer related genes/ miRNAs	-TF-miRNA regulatory interactions<br>-Network analysis<br>-Disease specific network<br>-Shortest paths between TF-miRNA pairs<br>-Identification of minimal regulatory modules	-Gene regulatory network and comprehensive network analysis	-Web service	-PITA<br>-PuTmiR

Supplementary Figures

Figure S1 – miRNA sample clustering and heatmap. (a) The heatmap visualization of the 19-dysregulated miRNAs. The miRNA expression values were normalized and scaled before visualization. (b) The PCA clustering for the normalized miRNA expression samples. The two highlighted PD samples are incorrectly clustered to the control cohort. They could be mislabelled and nevertheless they had negligible impact on the analysis when we excluded them.

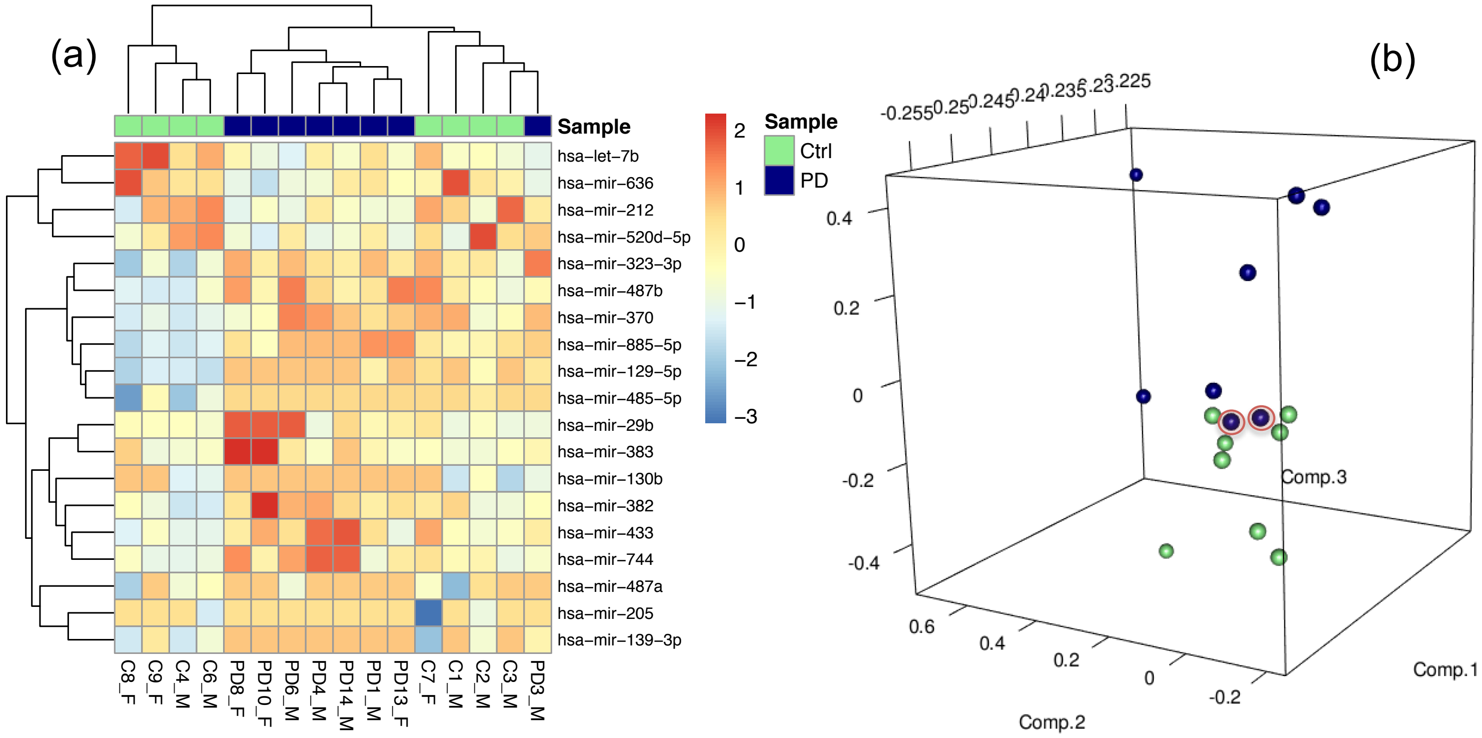

Figure S2 – The merged visualization of motifs A and B in the PD-GRN network. (a) The merged motif and (b) its functional homogeneity plot depicting the cumulative distribution of GO functional semantic scores of gene pairs of co-regulated genes in the examined motif (red) versus randomly selected genes (black). The p-value was calculated using the Kolmogorov- Smirnov test. The network motifs were visualized using the Cytoscape tool.

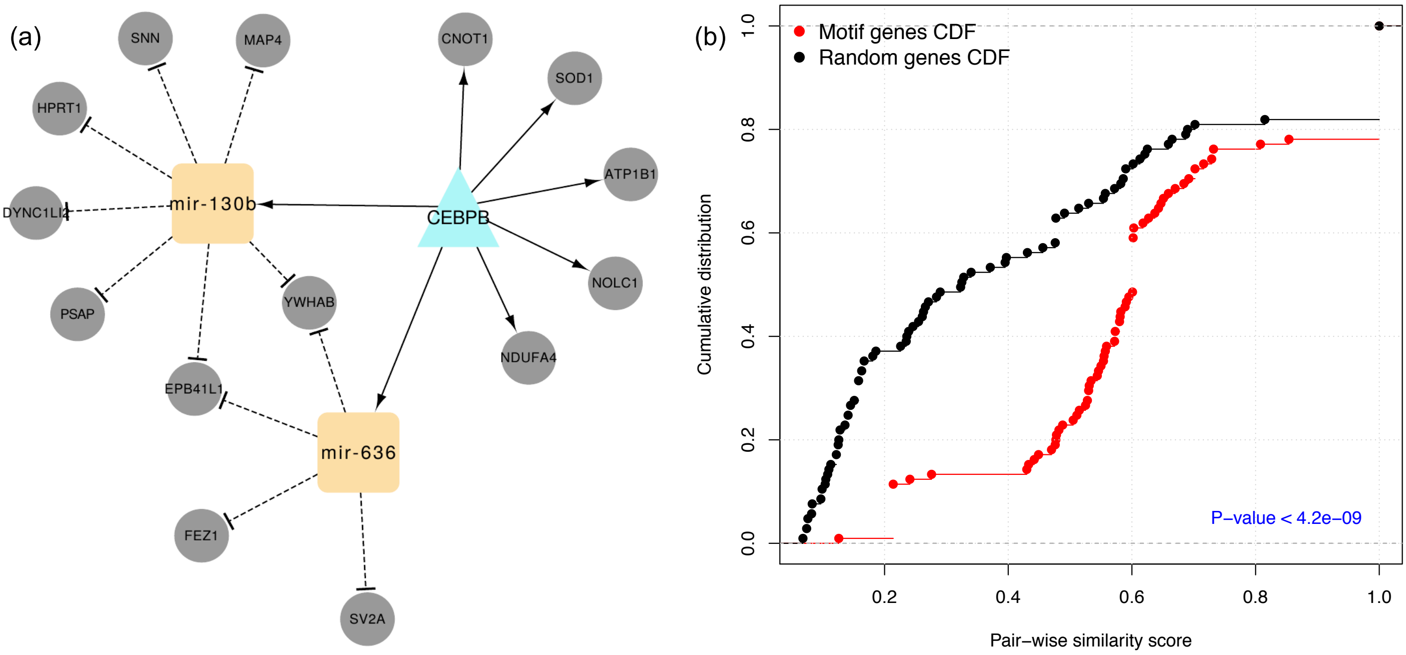

Figure S3 – Rank-Rank-Hypergeometric Overlap (RRHO) maps for the top predicted 16 Cmap drugs that reverse the whole PD expression signatures. The X- and Y-axis correspond to the ranked inverted gene expression profile for PD as described in the main text (from most downregulated to most upregulated), and to the Cmap gene expression profile indicated in the title of each map (ranked from most upregulated to most downregulated), respectively. Each data point x,y represents the significance of the overlap of two gene lists: the top-ranked genes from the PD profile up to rank x and the top-ranked genes from the Cmap profile up to rank y; the magnitude of the significance is indicated using a color scheme, with red and green indicating high and low significance, respectively. Patterns like those seen in the panels, with red dominating in the lower left corner and lower intensities elsewhere indicate considerable overlap between the genes at the top of both gene lists.

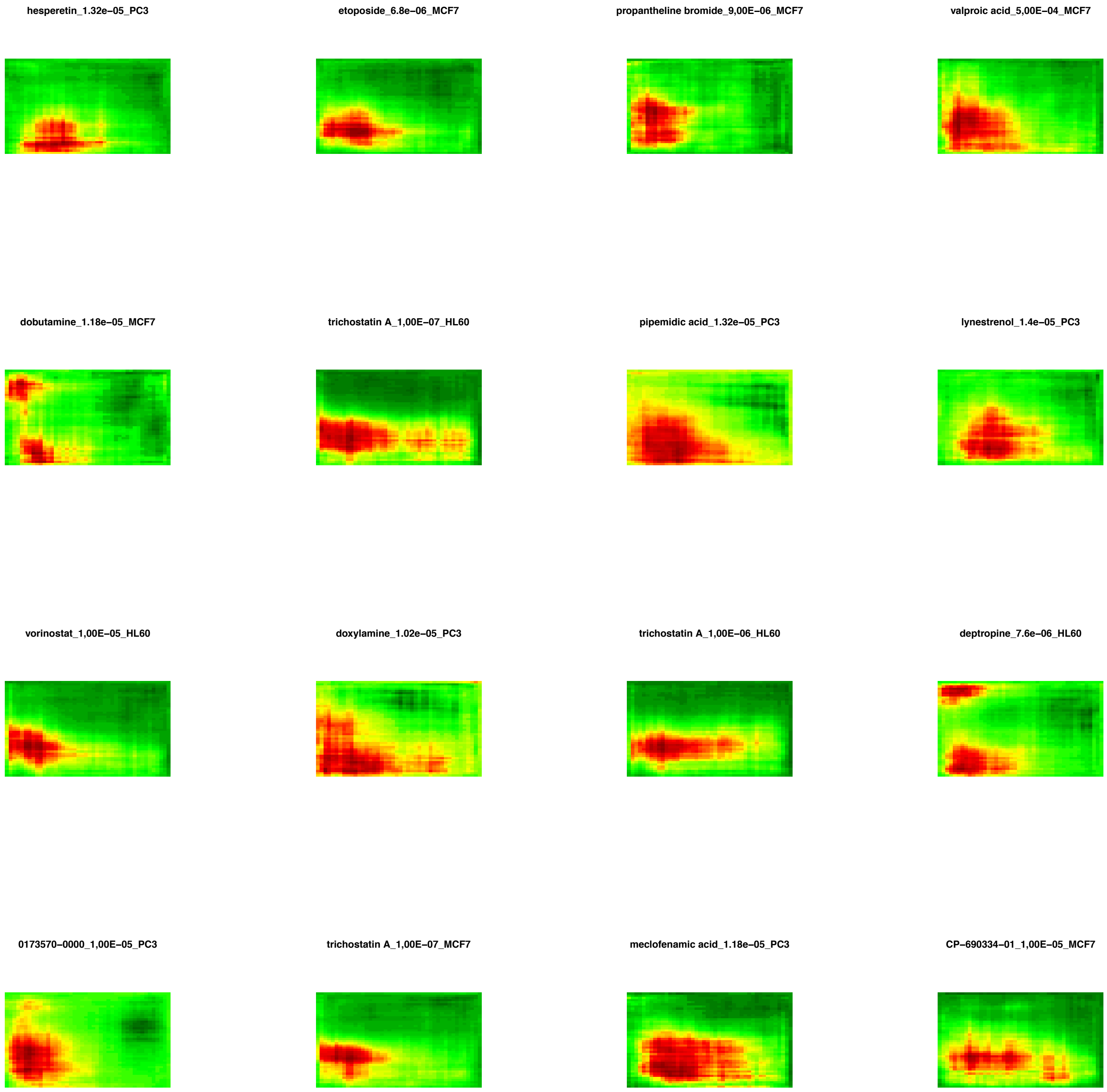

Supplement: Supplementary file 1 — Supplementary [file 41598_2018_25754_MOESM1_ESM.pdf]
